# Suppressing Cyanobacteria with Hydrogen Peroxide Is More Effective at High Light Intensities

**DOI:** 10.3390/toxins12010018

**Published:** 2019-12-31

**Authors:** Tim Piel, Giovanni Sandrini, Emily White, Tianshuo Xu, J. Merijn Schuurmans, Jef Huisman, Petra M. Visser

**Affiliations:** Department of Freshwater and Marine Ecology, Institute for Biodiversity and Ecosystem Dynamics, University of Amsterdam, P.O. Box 94240, 1090 GE Amsterdam, The Netherlands; t.f.piel@uva.nl (T.P.); giovanni.sandrini@gmail.com (G.S.); ewhite14@msn.com (E.W.); xutianshuo2012@126.com (T.X.); J.M.Schuurmans@uva.nl (J.M.S.); J.Huisman@uva.nl (J.H.)

**Keywords:** hydrogen peroxide, *Microcystis aeruginosa*, toxic cyanobacteria, microcystin, photosynthesis

## Abstract

Hydrogen peroxide (H_2_O_2_) can be used as an emergency method to selectively suppress cyanobacterial blooms in lakes and drinking water reservoirs. However, it is largely unknown how environmental parameters alter the effectiveness of H_2_O_2_ treatments. In this study, the toxic cyanobacterial strain *Microcystis aeruginosa* PCC 7806 was treated with a range of H_2_O_2_ concentrations (0 to 10 mg/L), while being exposed to different light intensities and light colors. H_2_O_2_ treatments caused a stronger decline of the photosynthetic yield in high light than in low light or in the dark, and also a stronger decline in orange than in blue light. Our results are consistent with the hypothesis that H_2_O_2_ causes major damage at photosystem II (PSII) and interferes with PSII repair, which makes cells more sensitive to photoinhibition. Furthermore, H_2_O_2_ treatments caused a decrease in cell size and an increase in extracellular microcystin concentrations, indicative of leakage from disrupted cells. Our findings imply that even low H_2_O_2_ concentrations of 1–2 mg/L can be highly effective, if cyanobacteria are exposed to high light intensities. We therefore recommend performing lake treatments during sunny days, when a low H_2_O_2_ dosage is sufficient to suppress cyanobacteria, and may help to minimize impacts on non-target organisms.

## 1. Introduction

Cyanobacteria can develop dense blooms in eutrophic lakes and reservoirs [1,2]. Many of the bloom-forming cyanobacteria, such as *Microcystis aeruginosa,* can produce secondary metabolites that are toxic to plants, invertebrates and vertebrates, including birds and mammals [3,4,5]. Toxic cyanobacteria can also be a human health risk, especially in bathing waters or drinking water supplies [6,7,8]. Consequently, toxic cyanobacterial blooms may interfere with the intake of water by drinking water companies, and lead to the closure of recreational lakes, sometimes with large socioeconomic consequences [9,10,11,12]. In view of the World Health Organization (WHO) guideline value for safe drinking water, it is essential to keep the concentrations of cyanotoxins such as microcystin low [4].

Eutrophication is one of the main driving forces behind the observed increase in cyanobacterial blooms worldwide [1,2,13,14]. Hence, the most sustainable solution to prevent bloom formation of toxic cyanobacteria is a reduction of nutrient inputs, particularly phosphorus (P) and nitrogen (N), into lakes and reservoirs [13,15,16]. However, due to nutrient storage and recycling in lake sediments, it often takes years and sometimes even decades before nutrient reduction efforts become successful [17,18,19]. Measures to reduce nutrient loss from agricultural land and to suppress runoff into surface waters could benefit farmers, and may help to improve the water quality of lakes and reservoirs [20,21,22]. However, the high nutrient demands of areas with extensive agriculture and large cities may remain a major obstacle to reduce nutrient inputs to sufficiently low levels to prevent cyanobacterial blooms.

If any long-term reduction of nutrient loads is not feasible, or has not yet resulted in the suppression of cyanobacterial blooms, emergency methods that can rapidly remove toxic cyanobacteria may be required to avoid problems with the intake of drinking water or bathing prohibitions [10,12,23,24,25]. Several emergency methods have been applied, but many of them have undesirable side effects, as they may involve the addition of metals (e.g., copper, aluminum) or persistent organic chemicals, or may result in the killing of non-target species, all of which severely affect the ecosystem [26,27,28].

Since several years, hydrogen peroxide (H_2_O_2_) has been used as an emergency method against cyanobacterial blooms [27,28,29]. H_2_O_2_ is a reactive oxygen species (ROS) that can cause oxidative stress and lead to cellular damage when present in sufficiently high concentrations [30,31]. H_2_O_2_ is formed naturally in surface waters by photochemical reactions when dissolved organic carbon is exposed to sunlight [32,33]. H_2_O_2_ is also produced by biological processes such as the Mehler reaction in eukaryotic photosynthesis [34,35] and a plethora of other metabolic processes [36,37]. In freshwater lakes, H_2_O_2_ is mostly degraded enzymatically by organisms [31,38], and also by redox-sensitive metal ions such as iron and manganese [39,40]. Natural H_2_O_2_ concentrations in the surface waters of lakes range from 1 to 46 µg/L (30–1570 nM) [33,40,41].

H_2_O_2_ selectively suppresses cyanobacteria because of the higher sensitivity of cyanobacteria to oxidative stress compared to eukaryotic phytoplankton [27,42,43,44,45]. The H_2_O_2_ concentration required to suppress bloom-forming cyanobacteria is in the range of 1–10 mg/L, depending on the species and environmental conditions [27,45], which range is several orders of magnitude higher than the natural H_2_O_2_ concentration in lakes, but below the H_2_O_2_ sensitivity threshold of most eukaryotic organisms [27,46,47]. Even though H_2_O_2_-degrading enzymes such as peroxiredoxins and thioredoxin-coupled peroxidases are present in cyanobacteria (e.g., Schuurmans et al. [48]), most cyanobacteria lack catalases and ascorbate peroxidases [49,50,51,52]. The lower H_2_O_2_ degradation capacity of cyanobacteria compared to eukaryotic algae might be traced back to a key difference in their photosynthetic physiology. Under high light stress, the chloroplasts of eukaryotic algae produce H_2_O_2_ in the Mehler reaction [34,35], whereas cyanobacteria use a different Mehler-like reaction involving flavodiiron proteins, where this reaction does not produce H_2_O_2_ [53,54,55,56]. As a consequence, eukaryotic phytoplankton tend to be better defended against low concentrations of H_2_O_2_ (1–10 mg/L), whereas cyanobacteria suffer severe oxidative damage under these conditions [27,42,43,57].

A key advantage of the use of H_2_O_2_ treatments to suppress cyanobacterial blooms is that the added H_2_O_2_ degrades to water and oxygen within a few days, and hence does not persist in the lake. However, to avoid oxidative damage to non-target species (e.g., heterotrophic and chemolithotrophic bacteria, zooplankton, macro-invertebrates), it is important to establish how the effectiveness of H_2_O_2_ treatments against cyanobacteria can be improved. One of the environmental variables that is likely to influence the effectiveness of H_2_O_2_ is light. Drábková et al. [46] investigated exposure of *Microcystis aeruginosa* to different H_2_O_2_ concentrations and found that high light intensities led to a stronger decline of the photosynthetic yield. Mikula et al. [58] reported that a low H_2_O_2_ dosage at high light could lead to an even stronger reduction of the photosynthetic yield than a high H_2_O_2_ dosage in darkness. However, neither of these studies investigated the effects of H_2_O_2_ and light intensity on the cyanotoxin microcystin (MC), which is of key interest in lake management. 

Moreover, in addition to light intensity, the underwater light color is also an important environmental variable that is likely to be of interest given the recent changes in water color by lake brownification [59,60,61]. Light color has a major impact on cyanobacterial photosynthesis [62] and hence may also affect the H_2_O_2_ treatment.

In this study, we assess how light intensity and light color influence the effectiveness of H_2_O_2_ against the microcystin-producing cyanobacterium *Microcystis aeruginosa* PCC 7806 (further named *Microcystis*). For this purpose, dense *Microcystis* cultures were pre-grown in laboratory chemostats provided with a nutrient-rich mineral medium and kept in steady state. Subsequently, samples from these chemostats were treated with a range of H_2_O_2_ concentrations exposed to different light intensities and light colors. Lakes with cyanobacterial blooms are often quite turbid (hazy). Therefore, we applied light intensities ranging from 0 to 150 µmol photons·m^−2^·s^−1^ as representative light conditions for the top 1–2 m of turbid lakes dominated by cyanobacterial blooms [63,64]. The effectiveness of the H_2_O_2_ treatments was evaluated by monitoring several physiological variables, including photosynthetic yield, MC concentrations and cell size. The results provide insight into how H_2_O_2_ damages cyanobacterial cells, and they also help to elucidate which light conditions are most suitable for the H_2_O_2_ treatment of cyanobacterial blooms in lakes.

## 2. Results

### 2.1. Photosynthetic Vitality

**Figure 1:**
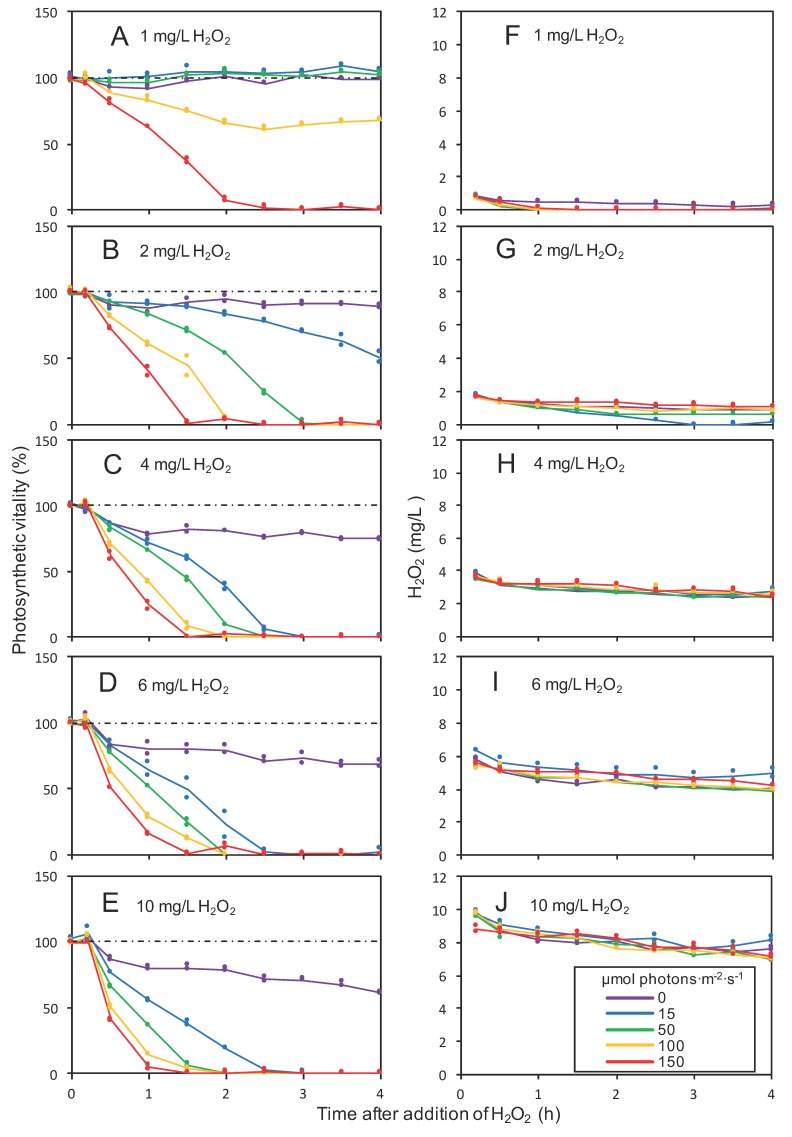
Effects of light intensity on photosynthetic vitality and H_2_O_2_ degradation after an addition of different H_2_O_2_ concentrations. The graphs show the decrease in (**A**–**E**) the photosynthetic vitality of *Microcystis* PCC 7806 and (**F**–**J**) H_2_O_2_ concentration, during the first 4 h after the addition of (**A**,**F**) 1 mg/L, (**B**,**G**) 2 mg/L, (**C**,**H**) 4 mg/L, (**D**,**I**) 6 mg/L and (**E**,**J**) 10 mg/L of H_2_O_2_. Photosynthetic vitality is defined as the photosynthetic yield of cells treated with H_2_O_2_ as a percentage of the control samples of cells not exposed to H_2_O_2_. Dots represent data of two independent biological replicates per treatment. Lines represent the average over the two biological replicates, where different colors represent different light intensities, and dashed black lines represent the control without H_2_O_2_.

The photosynthetic vitality of *Microcystis*, defined as the photosynthetic yield as percentage of the control treatment, was reduced by the H_2_O_2_ treatments (Figure 1A–E; Table S1). The decrease in photosynthetic vitality was accompanied by an increase of F_0_, followed by an increase of F_m_ after the first 1–2 h (Figure S1).

The decline of the photosynthetic vitality was strongly dependent on both the H_2_O_2_ dosage and the light intensity. H_2_O_2_ treatments in the dark had the smallest effect, whereas increasing light intensities strengthened the effect of H_2_O_2_ on the photosynthetic vitality. For example, an addition of 10 mg/L H_2_O_2_ in the dark caused only a 30% decline in photosynthetic vitality (Figure 1E), whereas the addition of 1 mg/L H_2_O_2_ at a light intensity of 150 µmol photons·m^−2^·s^−1^ caused a complete decline of the photosynthetic vitality to zero within 2.5 h (Figure 1A).

**Figure 2:**
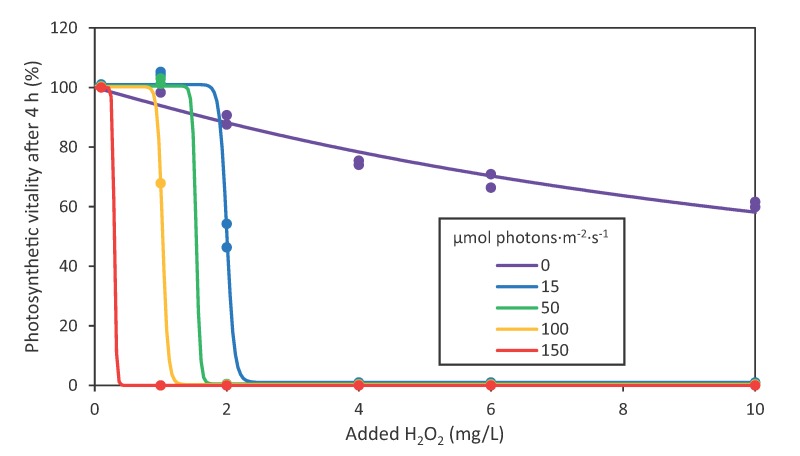
Dose–response models of the photosynthetic vitality after 4 h as function of the added H_2_O_2_ concentration. Dots represent the data of two independent biological replicates per H_2_O_2_ concentration. The colored lines represent the dose–response models of the photosynthetic vitality of *Microcystis* as function of the H_2_O_2_ concentration fitted with nonlinear regression for each light intensity (*n* = 12 data points per light intensity).

Dose-response models of the photosynthetic vitality after 4 h were fitted as function of the H_2_O_2_ concentration using nonlinear regression (Figure 2). The parameter estimates of the dose–response models are reported in Table S2. Pairwise comparison of the dose–response models using the Akaike Information Criterion corrected for small sample sizes (AICc) showed that the dose–response models differed between the different light intensities (Table S2). In other words, light intensity affected the H_2_O_2_ sensitivity of *Microcystis*. The estimated EC_50_ values ranged from >10 mg/L in darkness to <1 mg/L at a light intensity of 150 µmol photons·m^−2^·s^−1^ (Figure 2; Table S2). 

### 2.2. H_2_O_2_ Degradation

H_2_O_2_ was slowly degraded during the first 4 h of the experiment, irrespective of light intensity (Figure 1F–J). Degradation rates varied only slightly between the different H_2_O_2_ concentrations. In the treatment with 10 mg/L of H_2_O_2_ in the presence of *Microcystis*, the H_2_O_2_ concentration had declined to 7.41 ± 0.50 mg/L (mean ± standard deviation (SD); *n* = 10) after 4 h (Figure 1J). For comparison, in the controls without *Microcystis* that were provided with 10 mg/L of H_2_O_2_, the H_2_O_2_ concentration declined only marginally, to 9.92 ± 0.28 mg/L (*n* = 10) and 9.74 ± 0.26 mg/L (*n* = 10) after 4 h in Milli-Q water and BG-11 medium, respectively. Hence, H_2_O_2_ was degraded significantly faster in the presence than in the absence of *Microcystis* cells (Student’s *t*-test of treatment vs control in BG-11 medium: t = 20.3, df = 9, *p* < 0.001).

### 2.3. Microcystin Concentrations

**Figure 3:**
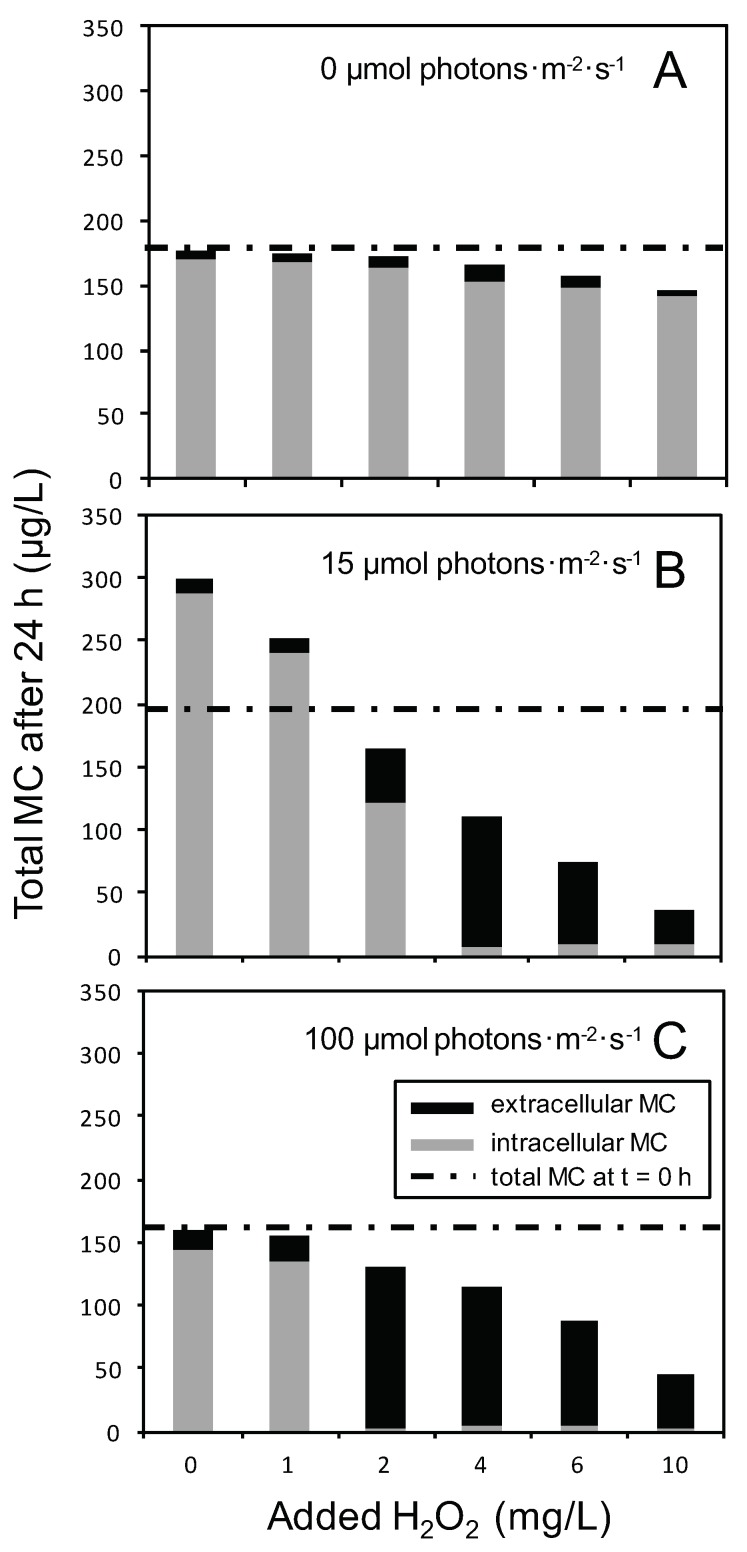
Total intracellular and extracellular microcystin (MC) concentrations 24 h after addition of different hydrogen peroxide (H_2_O_2_) concentrations. *Microcystis* PCC 7806 was exposed to three different light intensities: (**A**) 0 µmol photons·m^−2^·s^−1^, (**B**) 15 µmol photons·m^−2^·s^−1^, and (**C**) 100 µmol photons·m^−2^·s^−1^. Gray and black bars show intracellular and extracellular MC, respectively, at 24 h after H_2_O_2_ addition. Each bar represents the average of two independent biological replicates per treatment. Dashed lines indicate the total MC concentration prior to H_2_O_2_ addition (t = 0 h).

The total MC concentration, as well as the proportion of MC in the cells (intracellular MC), and in the water phase (extracellular MC), was strongly affected by both the H_2_O_2_ dosage and light intensity (Figure 3). After 24 h exposure to H_2_O_2_ in the dark, the intracellular MC of *Microcystis* cells had decreased only slightly, and the extracellular MC concentration was only a minor fraction of the total MC concentration, even at the highest H_2_O_2_ dosage of 10 mg/L (Figure 3A). The intracellular MC had increased in *Microcystis* cells treated with 0 and 1 mg/L of H_2_O_2_ at 15 µmol photons·m^−2^·s^−1^ (Figure 3B). Most likely, oxidative stress at this low light level and H_2_O_2_ dosage was minimal, which enabled cell growth, and hence an increase of the MC concentration during the 24-h incubation. In contrast, hardly any intracellular MC could be detected after 24 h, and most MC ended up in the extracellular MC pool when *Microcystis* cells were treated with ≥4 mg/L H_2_O_2_ at 15 µmol photons·m^−2^·s^−1^ (Figure 3B), or when they were treated with ≥2 mg/L H_2_O_2_ at 100 µmol photons·m^−2^·s^−1^ (Figure 3C).

In those H_2_O_2_ treatments where the intracellular MC disappeared, the extracellular MC concentration measured after 24 h of H_2_O_2_ exposure did not reach the total MC concentration of the *Microcystis* cultures prior to H_2_O_2_ exposure. This indicates that part of the MC was lost or not recovered by our analysis.

As a control, we exposed pure MC-LR in BG-11 medium, but without *Microcystis* cells to the different H_2_O_2_ and light treatments. The results showed that pure MC-LR was hardly degraded by H_2_O_2_ irrespective of the light conditions; the MC-LR concentration declined by, on average, less than 1.5% after 24 h (Figure S2).

### 2.4. Biovolume, Cell Size and Cell Counts

**Figure 4:**
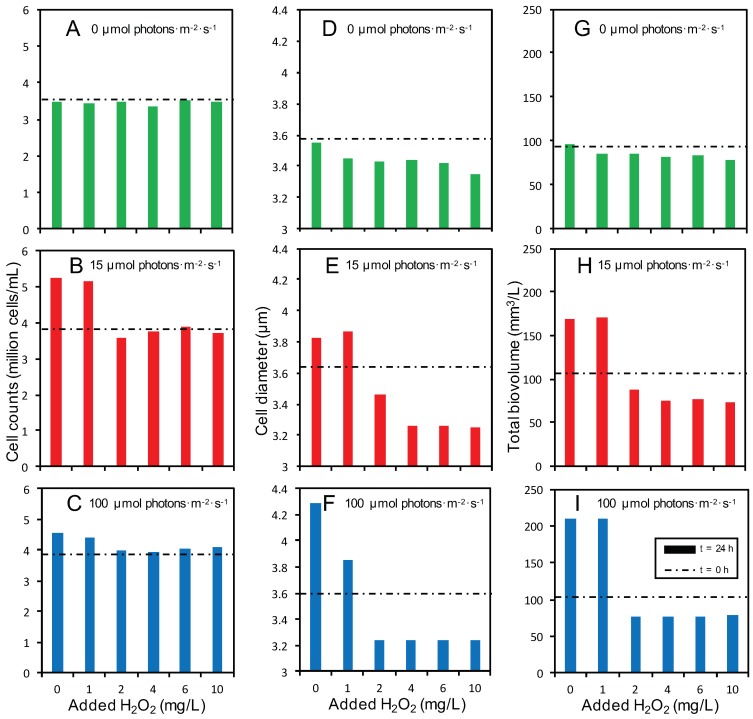
Cell counts (**A**–**C**), cell diameter (**D**–**F**) and total biovolume (**G**–**I**) of *Microcystis* PCC 7806 at 24 h after addition of different H_2_O_2_ concentrations. *Microcystis* PCC 7806 was exposed to H_2_O_2_ at three different light intensities: (**A**,**D**,**G**) 0 µmol photons·m^−2^·s^−1^, (**B**,**E**,**H**) 15 µmol photons·m^−2^·s^−1^ and (**C**,**F**,**I**) 100 µmol photons·m^−2^·s^−1^. The bars represent the average of two independent biological replicates per treatment at 24 h after H_2_O_2_ addition. For comparison, horizontal, dashed lines indicate the cell counts (**A**–**C**), cell diameter (**D**–**F**) and total biovolume (**G**–**I**) prior to H_2_O_2_ addition (t = 0 h).

Cell numbers increased during the 24-h time span of our experiments when *Microcystis* was grown in the light at 0 and 1 mg/L H_2_O_2_, whereas cell numbers remained constant for all H_2_O_2_ treatments in the dark and for cells exposed to ≥2 mg/L H_2_O_2_ in the light (Figure 4A–C). The constant cell numbers in the treatments exposed to ≥2 mg/L H_2_O_2_ indicate that these cells had ceased growth, but did not disintegrate during the 24-h experiments of this study, as confirmed by a microscopic inspection of the samples. Interestingly, the diameter of *Microcystis* cells was slightly reduced when they were exposed to ≥2mg/L H_2_O_2_ in the light (Figure 4D–F). As a consequence of the observed changes in cell numbers and cell size, the total biovolume of *Microcystis* grown in the light was much higher at 0 and 1 mg/L H_2_O_2_ than at ≥2 mg/L H_2_O_2_ (Figure 4G–I). 

### 2.5. Light Color

**Figure 5:**
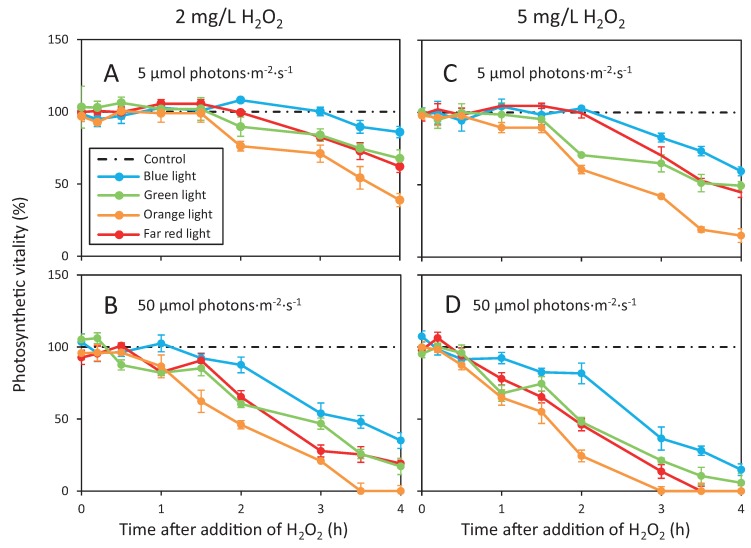
Effects of different light colors on the photosynthetic vitality after addition of H_2_O_2_. The graphs show the decrease in photosynthetic vitality of *Microcystis* PCC 7806 in blue (450 nm), green (530 nm), orange (630 nm) and far red light (730 nm) after H_2_O_2_ addition of (**A**,**B**) 2 mg/L and (**C**,**D**) 5 mg/L. For each light color, *Microcystis* was exposed to a light intensity of (**A**,**C**) 5 µmol photons·m^−2^·s^−1^ and (**B**,**D**) 50 µmol photons·m^−2^·s^−1^. Photosynthetic vitality is defined as the photosynthetic yield of cells treated with H_2_O_2_ as a percentage of the control samples of cells not exposed to H_2_O_2_. Symbols represent the average ± standard deviation (SD) of four biological replicates. Dashed black lines represent the control without H_2_O_2_.

Light color also affected the response of *Microcystis* cells to H_2_O_2_ (Figure 5). Orange light (630 nm) is mostly absorbed by phycocyanin in the phycobilisomes of *Microcystis*. H_2_O_2_ exposure in orange light consistently resulted in the strongest decline of the photosynthetic vitality, irrespective of the H_2_O_2_ dosage and light intensity. Blue light (450 nm) is absorbed by Chl *a* in the photosystems and by carotenoids. H_2_O_2_ exposure in blue light consistently had the weakest effect of the four colors investigated. For example, in orange light of 50 µmol photons·m^−2^·s^−1^ the addition of 2 mg/L H_2_O_2_ was sufficient to cause a complete collapse of the photosynthetic vitality (Figure 5B), whereas in blue light of the same light intensity even exposure to 5 mg/L H_2_O_2_ did not result in a complete decline of the photosynthetic vitality (Figure 5D). Green light (530 nm) and far-red light (730 nm) are less absorbed by the photosynthetic pigments of *Microcystis* cells than orange and blue light. The response of the photosynthetic vitality to H_2_O_2_ addition in green and far-red light was intermediate between the response in orange and the response in blue light.

## 3. Discussion

### 3.1. Light Enhances Effectiveness of H_2_O_2_

Suppressing cyanobacteria in lakes with H_2_O_2_ should be done with a concentration that is effective against cyanobacteria, but minimizes harmful effects to nontarget organisms such as zooplankton [27]. Our study shows that environmental conditions can influence the effectiveness of H_2_O_2_ treatments. Specifically, our results demonstrate that the H_2_O_2_ effectiveness against cyanobacteria is drastically enhanced at high light intensities. Even a very low H_2_O_2_ concentration of 1 mg/L, which hardly showed any effect at darkness or low light, was fatal to *Microcystis* under high light conditions. Consequently, days of long and intense sunshine should be selected to enhance the efficiency when treating cyanobacteria in lakes.

Possibly, preconditioning to high light may improve the defense of cyanobacteria against oxidative stress, and thereby may have an effect on the sensitivity of *Microcystis* to H_2_O_2_. For instance, previous exposure to high light influenced the response of the marine cyanobacterium *Synechococcus* sp. WH7803 to oxidative stress [65]. Furthermore, experiments with *Microcystis* PCC 7806 have shown that exposure to oxidative stress leads to a binding of microcystins to proteins to protect them against oxidative stress [66], and triggers the production of anti-oxidant enzymes such as peroxiredoxins and peroxidases [48]. 

However, the *Microcystis* cultures of our study were pre-grown at relatively low light intensities (~15 μmol photons·m^−2^·s^−1^), yet the observed sensitivity to H_2_O_2_ was similar as in Mikula et al. [58] and Lürling et al. [67], who cultured *Microcystis* at higher light intensities of 140 µmol photons·m^−2^·s^−1^ and 100 µmol photons·m^−2^·s^−1^, respectively. Moreover, Drábková et al. [46] specifically tested if pre-conditioning to high light intensities of the same *Microcystis* strain as in our study influenced its H_2_O_2_ sensitivity, but then concluded that this was not the case.

### 3.2. Damage Caused by H_2_O_2_

Why is H_2_O_2_ more effective against cyanobacteria at high light intensities? How H_2_O_2_ damages cyanobacterial cells is not yet completely understood. It is known that H_2_O_2_ damages the D1 protein [68]. The D1 protein is part of the reaction center of photosystem II (PSII), and is in direct contact with the oxygen-evolving manganese cluster. The D1 protein and PSII are commonly damaged by light absorption, even in the absence of reactive oxygen species (ROSs) [69]. This damage can be remedied because the D1 protein has a very high turnover rate, and under modest light conditions the repair of PSII is faster than the damage rate, so that photoinhibition can usually be avoided [65,70]. However, H_2_O_2_ may cause aggregation of the D1 proteins [68] and it also disrupts the D1 repair mechanism [71,72,73], which makes cells more sensitive to photoinhibition. Moreover, this interactive effect between H_2_O_2_ and photoinhibition will be stronger at high light intensities, in agreement with our observations.

Similar to previous studies, the effectiveness of H_2_O_2_ addition was assessed by measurements of the photosynthetic yield using PAM fluorometry (e.g., [27,45,46,58]). The photosynthetic yield reflects the efficiency of photosynthetic electron transport at PSII, and hence will be very sensitive to the damage of PSII and the D1 protein induced by H_2_O_2_. The photosynthetic yield is derived from two fluorescence values, F_0_ and F_m_ [74,75]. F_0_ is the minimum fluorescence of a dark-adapted culture, which can be interpreted as the background fluorescence when the reaction centers of PSII are “open”. F_m_ is the maximum fluorescence measured after a saturating light pulse when all reaction centers of PSII are “closed”, and thus, no further electrons can be accepted and all additional energy is dissipated as heat and fluorescence [75]. Accordingly, an increase in damaged D1 proteins is expected to reduce the amount of open reaction centers, which will increase F_0_ and consequently will reduce the photosynthetic yield. This was indeed observed in our study (Figure S1).

Our observation that H_2_O_2_ had a stronger effect on the photosynthetic yield in orange light (630 nm) than in the more energetic blue light (450 nm) indicates that the damaging effect of H_2_O_2_ is linked to the absorption of light by different photosynthetic pigments. While Chl *a* and carotenoids are responsible for absorption of blue light, the chromophore phycocyanobilin in the phycobilisomes of *Microcystis* absorbs orange light. Luimstra et al. [62,76] investigated the effect of orange and blue light on cyanobacterial photosynthesis, and found that orange light leads to much higher rates of oxygen production by PSII than blue light. They hypothesized that the reason for this difference in oxygen evolution rates is caused by the way orange and blue light are distributed over PSI and PSII. More specifically, cyanobacteria have much more of their Chl *a* in PSI than in PSII, and hence, blue light is mostly transferred to PSI [62,77,78,79]. Conversely, most phycobilisomes are attached to PSII under normal growth conditions (e.g., [62,80]), and hence the absorbed orange light is mostly transferred to PSII. As a result, the relative amount of light energy that arrives at PSII, where H_2_O_2_ damages the D1 protein, is highest in orange light and lowest in blue light.

In addition to the effects of H_2_O_2_ on photosynthesis, H_2_O_2_ and other reactive oxygen species (ROSs) may also cause further cellular damage [30,81]. Our H_2_O_2_ treatments caused bleaching of *Microcystis*, but did not result in a complete disintegration of the cells, as cell counts were stable throughout the 24-h experiments of this study, as well as in the 120-h experiments of Schuurmans et al. [48]. However, cell sizes of *Microcystis* and therefore also the total cyanobacterial biovolume were negatively affected by the H_2_O_2_ treatments. The decrease in cell size is likely caused by a leakage of the cellular contents. 

Several studies found that cells open and die upon H_2_O_2_ exposure, as shown by SYTOX staining of cells with damaged cell membranes [58], electron microscopy [68,82,83] and measurement of K^+^ release [83], as well as caspase-3-like activity as a trigger for programmed cell death [82,83]. Perforation of the cell membrane would also explain the release of intracellular MC, as observed in our study and several previous studies [67,84,85].

### 3.3. Microcystin Dynamics

The decrease of intracellular MC induced by the H_2_O_2_ treatments of ≥2 mg/L H_2_O_2_ at 15 and 100 µmol photons·m^−2^·s^−1^ was accompanied by a parallel increase of extracellular MC (Figure 3). Yet, the total MC concentration (i.e., the sum of intracellular and extracellular MC) prior to H_2_O_2_ addition could not be recovered within 24 h after H_2_O_2_ addition, a phenomenon also observed by Lürling et al. [67] and Schuurmans et al. [48]. The disappearance of MC cannot be attributed to H_2_O_2_-mediated degradation of MC, since pure MC in BG-11 medium was hardly degraded by H_2_O_2_ (Figure S2). However, it is known that MC binds to proteins when cells are exposed to oxidative stress, for instance, by H_2_O_2_ addition [66]. Protein-bound MC is not methanol extractable, and hence will not be detected by the standard methods of MC analysis [86]. In other words, most likely, some part of the MC was temporarily bound to cellular proteins in response to the oxidative stress induced by H_2_O_2_ [66], and therefore was no longer visible in our analysis. Schuurmans et al. [48] showed that 120 h after H_2_O_2_ addition, the total extracellular MC concentration approached the total intracellular MC at the start of the experiments, which indicates that the protein-bound MC was gradually released in the mineral medium after further disintegration of the cells.

The fate of released MC is of relevance for the application of H_2_O_2_ treatments in lakes and drinking water reservoirs. The absence (or near absence) of bacteria capable of the microbial degradation of MC explains why extracellular MC concentrations remained relatively high after H_2_O_2_ addition in our laboratory experiments. In contrast, aquatic microbial communities of lakes and sediments can break down MC released by cyanobacterial blooms [87,88,89,90]. While new pathways for bacterial MC degradation are still being discovered [91,92,93], recent studies have shown that complete MC degradation in the field can occur within a few days [94,95,96]. This is consistent with the lake treatment of Matthijs et al. [27], where MC disappeared in 1–2 days after H_2_O_2_ addition. In addition, H_2_O_2_ treatments supplemented with the MC-degrading enzyme Mlr-A can further enhance the degradation of MC released by lysing cyanobacteria [97]. Whether interactions between H_2_O_2_ and high light intensities affect this microbial degradation of MC is an interesting open question that may deserve further attention in future studies.

### 3.4. Implications for Lake Treatments

Our results show that light enhances the toxicity of H_2_O_2_ for cyanobacteria, as H_2_O_2_-treated cells exposed to higher light intensities have lower photosynthetic yields, shrink in size, and release MC. One might argue that light intensities in our laboratory experiments (≤150 µmol photons·m^−2^·s^−1^) were much lower than the incident sunlight at the surface of lakes during sunny days (up to 2000 µmol photons·m^−2^·s^−1^; e.g., [64]). However, light intensities at the lake surface can be lowered by ~90% due to cloud cover, and decrease rapidly with depth depending on the transparency of the lake [98]. Bloom-forming cyanobacteria often become dominant in relatively turbid lakes, and cyanobacterial blooms themselves even further reduce the transparency, such that light intensities ≤150 µmol photons·m^−2^·s^−1^ are reached within the first 1–2 m, and sometimes within the first few decimeters, of the water column even during sunny days (e.g., [63,64]). Hence, the range of light intensities applied in our study are highly relevant for many cyanobacterial blooms.

In stagnant waters under calm weather conditions, however, buoyant cyanobacteria such as *Microcystis* spp. may float upwards to the surface and form dense surface scums [2,99]. In this case, the top layer of the scum will be exposed to full sunlight and may suffer from severe photoinhibition [100]. 

Extrapolation of our results suggests that interference of H_2_O_2_ with the D1 repair mechanism is expected to lead to an even stronger suppression of the PSII activity at these high light intensities. This is supported by studies at Lake Taihu and Lake Dianchi, in China [101,102], where these interactive effects between H_2_O_2_ and light intensity were observed in samples taken from cyanobacterial blooms. In these studies, the samples were transferred to beaker glasses, exposed to H_2_O_2_ and placed in full sunlight, which resulted in a much stronger reduction of the photosynthetic yield, compared to control samples that were placed in the dark or shaded.

Light intensity did not have a major effect on the degradation rate of H_2_O_2_ in our laboratory experiments. However, degradation of H_2_O_2_ in lakes is usually much faster than the low H_2_O_2_ degradation rates in our axenic *Microcystis* cultures [27]. In lakes, anti-ROS enzymes of the microbial community, including bacteria as well as eukaryotic phytoplankton, and oxidation–reduction reactions with organic matter and metal ions are largely responsible for the degradation of H_2_O_2_ [38,41,45]. Furthermore, photochemical production of H_2_O_2_ and H_2_O_2_-mediated photodegradation of organic matter are known to have major impact on the H_2_O_2_ dynamics in lakes [38,40]. Hence, contrary to our lab experiments, light intensity may affect H_2_O_2_ degradation in lakes, which may influence the exposure time of cyanobacteria to the H_2_O_2_ treatment.

Our results show that light color has an effect as well, as *Microcystis* was more sensitive to H_2_O_2_ in orange than in blue light. These results may imply that it can be difficult to effectively suppress an upcoming cyanobacterial bloom in clear, blue lakes. In most cases, however, cyanobacterial blooms occur in more turbid lakes, where green or orange-red light often dominates the underwater light field [61]. In particular, lakes with relatively high concentrations of dissolved organic carbon (DOC) tend to be predominated by orange wavelengths that are effectively captured by the phycocyanobilin-pigments of bloom-forming cyanobacteria [62,103]. For example, Stomp et al. [104] reports that Lake Groote Moost, a turbid peat lake with high DOC concentrations, is characterized by an orange-red underwater spectrum, and is often dominated by cyanobacterial blooms. Although the high turbidity of these peat lakes reduces the overall light intensity, the remaining orange underwater color may still provide a suitable light spectrum to effectively suppress cyanobacterial blooms with H_2_O_2_.

Extrapolation of our laboratory results to lake conditions can help optimizing H_2_O_2_ treatments of cyanobacterial blooms. Our results imply that lake treatments would require less H_2_O_2_ on sunny days than on cloudy days. Similarly, lake treatments will be much more effective when H_2_O_2_ is administered during daytime than in the evening or at night. Furthermore, depending on the transparency of the water column, a surface bloom could be treated with a lower H_2_O_2_ concentration than a bloom located deeper in the water column or near the thermocline. Treating cyanobacterial blooms with H_2_O_2_ under the most effective light conditions may help to reduce the dosage, and hence the cost of the treatment, and may diminish potential side effects on other organisms.

## 4. Materials and Methods

### 4.1. Chemostat Cultures

The axenic strain *Microcystis aeruginosa* strain PCC 7806 was kindly provided by the Pasteur Culture Collection (Paris, France). *Microcystis* was pre-cultured in four chemostats specially designed for phytoplankton growth [105] to ensure that all *Microcystis* experienced the same growth conditions prior to the hydrogen peroxide (H_2_O_2_) experiments. Once the chemostats reached steady state, samples from the chemostats were transferred to batch culture experiments with H_2_O_2_ additions as described below.

Each chemostat consisted of a flat culture vessel that was illuminated from one side by white fluorescent tubes (Philips Master TL-D 90 De Luxe 18 W/965; Philips Lighting, Eindhoven, The Netherlands). The incident irradiance (*I_in_*) was set at 15 μmol photons·m^−2^·s^−1^ measured with a LI-COR LI-250 quantum photometer (LI-COR Biosciences, Lincoln, NE, USA). *Microcystis* was cultured in the nutrient-rich BG-11 medium [106] with 10 mmol/L NaNO_3_. The dilution rate of the chemostats was set at 0.01 h^−1^. 

The chemostats had an optical path length of 5 cm and an effective working volume of approximately 1.8 L. The temperature in the chemostats was maintained at 25 °C using stainless steel cooling fingers connected to two water baths (Haake A28F/AC200; Thermo Fisher Scientific, Pittsburgh, PA, USA). This temperature is representative for surface waters dominated by cyanobacterial blooms during warm summers at our latitude [64,107]. The chemostats were aerated with pressurized air containing 400 ppm CO_2_ at a flow rate of 30 L/h, as described by Sandrini et al. [108].

Regular microscopical observations of chemostat samples did not reveal contamination with other photosynthetic organisms. Although the chemostats were started with axenic cultures of *Microcystis*, we could not prevent contamination by a few heterotrophic bacteria during the chemostat experiments. At the end of the experiments, the number of heterotrophic bacteria in the samples was <<5% of the total cell counts.

### 4.2. H_2_O_2_ Toxicity Tests in Batch Cultures

To expose *Microcystis* to different concentrations of H_2_O_2_, 250 mL samples from the four steady-state chemostats were mixed and diluted with ~2000 mL sterile BG-11 mineral medium to reach a total *Microcystis* biovolume of 100 mm^3^/L in 3 L of mineral medium. This stock culture was used to carry out batch culture experiments in 12 Nalgene plastic bottles (Thermo Fisher Scientific, Pittsburgh, PA, USA) of 250 mL each and without lids. Each bottle received 200 mL of culture and was aerated with compressed air. The temperature was maintained at 25 °C using stainless steel cooling fingers connected to a water bath. H_2_O_2_ concentrations added to the batch cultures of *Microcystis* were 0, 1, 2, 4, 6 and 10 mg/L. Each H_2_O_2_ treatment was performed in duplicate. In addition, 10 mg/L of H_2_O_2_ was added to two plastic bottles with sterilized BG-11 medium and two plastic bottles with Milli-Q water (Merck KGaA, Darmstadt, Germany), but without *Microcystis*, to serve as controls for the degradation rate of H_2_O_2_ without cells.

We repeated this experiment five times, each time at a different light intensity. The time interval between experiments at different light intensities was about two weeks, to enable recovery of the chemostats to steady state after sampling. Light was provided by white fluorescent tubes (Philips Master TL-D 90 De Luxe 18 W/965, Philips Lighting, Eindhoven, The Netherlands) with incident light intensities of 0, 15, 50, 100 and 150 µmol photons·m^−2^·s^−1^ set by neutral density filters (LEE Filters, Andover, UK) and the distance between the fluorescent tubes and batch cultures. This range of light intensities is commonly found in the first 1–2 m of turbid lakes dominated by cyanobacteria [63,64]. The light spectrum of the white fluorescent tubes covers the entire range of photosynthetically active radiation (PAR, 400–700 nm), but differs somewhat from sunlight, as it has several distinct peaks at, e.g., 435, 489, 545, 589 and 612 nm.

Samples (5 mL) were taken just before adding H_2_O_2_, immediately after adding H_2_O_2_, and every half hour after adding H_2_O_2_ for a total of 4 h for the analysis of the photosynthetic vitality and H_2_O_2_ concentration. Furthermore, 5 mL samples were taken just before adding H_2_O_2_ and 24 h after adding H_2_O_2_ for analysis of MC concentrations and cell numbers. Cell numbers and cell volumes were determined using a Casy 1 TTC cell counter with a 60 μm capillary (Schärfe System GmbH, Reutlingen, Germany). Cell size was calculated from cell volume assuming a spherical shape of the cells.

### 4.3. Photosynthetic Vitality

The 5 mL samples were filtered using 1.2 μm pore size 25 mm GF/C filters (Whatman GmbH, Dassel, Germany) placed on a Millipore 1225 Sampling Manifold (Merck KGaA, Darmstadt Germany). The cells on the filters were dark-adapted for 5 min by covering the filters with rubber stoppers on the Millipore filtration unit. The potential photosynthetic yield of the cells was determined using a Mini-PAM fluorometer (Walz, Effeltrich, Germany). The instrument was equipped with an 80 cm long glass fiber cable, mounted in a rubber stopper of the Millipore filtration unit. 

After dark adaptation, the rubber stoppers were exchanged with the rubber stopper with the glass fiber cable and the fluorescence measurements were started. The photosynthetic yield (ϕPSII, also known as the quantum yield of PSII electron transport) was calculated according to Genty et al. [109]:

(1) $$\phi PSII=\left(F_{m}-F_{0}\right)/F_{m}$$

where *F*_0_ and *F_m_* are the minimum and maximum fluorescence, respectively. The photosynthetic vitality is defined as the photosynthetic yield of cells treated with H_2_O_2_ as a percentage of the photosynthetic yield of cells from control samples not exposed to H_2_O_2_.

### 4.4. Dose-Response Models

The photosynthetic vitality (PV) was described as function of the added H_2_O_2_ concentrations, according to the following logistic dose–response model:

(2) $$PV=\frac{C}{1+e^{B\left(log_{10}\left[H_{2}O_{2}\right]-log_{10}\left(A\right)\right)}}$$

where *A* is the estimated EC_50_ value (i.e., the H_2_O_2_ concentration at which *PV* is 50%), *B* is the slope of the dose–response model and *C* is the photosynthetic vitality of the control samples not exposed to H_2_O_2_ (i.e., *C* was set to 100%). The parameters A and B of the dose–response model were estimated by fitting the model to the photosynthetic vitality measured after 4 h using nonlinear regression, which minimizes the residual sum of squares using an iterative Levenberg–Marquardt algorithm.

First, a dose–response model was fitted for each light intensity separately. Subsequently, we applied a pairwise comparison to test whether the PV data of two adjacent light intensities is best described by these two separate dose–response models, or by a single dose–response model that captures the PV data of both light intensities. Model selection was based on the Akaike Information Criterion (AIC). More specifically, we used a modified version of the AIC known as the AICc, which corrects for small sample size according to:

(3) $$\mathrm{AICc}=2k+n\mathrm{LN}\left(RSS\right)+\left(\frac{2k^{2}+2k}{n-k-1}\right)$$

where *k* is the number of parameters, *n* the number of data points and *RSS* the residual sum of squares of the model. Accordingly, *k* = 4 if the PV data of two adjacent light intensities can best be described by two separate dose–response models, whereas *k* = 2 if a single dose–response model can be used. The approach with the smaller AICc value better describes the data.

### 4.5. Hydrogen Peroxide Analysis

Filtrates obtained from the filtered samples (see Section 4.3) were used for analysis of the H_2_O_2_ concentration. A small amount of filtrate (100 μL) was mixed with 100 μL of 2 mmol/L *p*-nitrophenylboronic acid reagent (Merck KGaA, Darmstadt, Germany) in a 96-well microtiter plate according to Lu et al. [110]. H_2_O_2_-dependent formation of *p*-nitrophenolate at room temperature was complete in 30 to 45 min, and the color remained stable for several hours. Absorption was measured at the absorption peak of *p*-nitrophenolate (405 nm) using a SPECTROstar Nano plate reader (BMG Labtech GmbH, Ortenberg, Germany). A calibration curve for H_2_O_2_ concentrations ranging from 0.01–10 mg/L was generated using a 33% (*w*/*w*) stock solution of H_2_O_2_ (VWR, Amsterdam, The Netherlands). For each filtrate, the H_2_O_2_ concentration was measured in quadruplicate. Pilot experiments showed that losses of H_2_O_2_ by the filtration procedure were <1%.

### 4.6. Microcystin Analysis and Degradation

To determine cellular MC contents and the MC concentrations in the water phase, the cells on the filters (defined as intracellular MC) and in the filtrates (extracellular MC) were stored at −20 °C and subsequently freeze-dried. The freeze-dried samples were resuspended, and MC was extracted in 1 mL of 75% MeOH according to Tonk et al. [111] and analyzed using high performance liquid chromatography (HPLC) according to Van de Waal et al. [112], using a Shimadzu LC-20AD HPLC system with a SPD-M20A photodiode array detector (Shimadzu, Kyoto, Japan). *Microcystis* PCC 7806 produces two MC variants, [Asp3]MC-LR and MC-LR [113], but peaks of the two MC variants could not be completely separated. Therefore, both peaks were summed and are referred to as MC in the following sections.

To investigate potential degradation of pure MC-LR by H_2_O_2_, this pure MC-LR (ENZO Life Science BVBA, Brussels, Belgium) was dissolved in 100% MeOH and diluted with BG-11 medium to a final concentration of ~230 µg/L of MC-LR in a total volume of 1.5 L. Subsequently, 100 mL samples were taken and exposed to different H_2_O_2_ concentrations at different light intensities using the same set-up as for the batch culture experiments described in Section 4.2.

### 4.7. Effect of Light Color

*Microcystis* samples were diluted to OD_750_ = 0.3 with freshly made, sterile BG-11 medium, and subsequently 3 mL aliquots were transferred to 12-well plates. We used a total of eight plates that were exposed to two different light intensities (5 or 50 µmol photons·m^−2^·s^−1^) of either blue light (450 nm), green light (530 nm), orange light (630 nm) or far red light (730 nm) provided by custom-made light emitting diode (LED) light panels (Technology Centre, University of Amsterdam, The Netherlands in collaboration with Philips Lightning B.V., Eindhoven, The Netherlands). The light spectra of the LEDs had a full width at a half maximum of ~20 nm, as confirmed with a RAMSES-ACC-VIS spectroradiometer (TriOS, Oldenburg, Germany). Light intensities were measured with a LI-COR LI-250 quantum photometer (LI-COR Biosciences, Lincoln, NE, USA).

On each 12-well plate, four wells served as control (0 mg/L H_2_O_2_), four other wells received 2 mg/L H_2_O_2_ and the four remaining wells received 5 mg/L H_2_O_2_. The photosynthetic vitality of the *Microcystis* cultures in the well-plates was monitored through the bottom glass of the wells for the first 4 h after H_2_O_2_ addition with a PAM fluorometer, as described in Section 4.3.

## Supplementary Materials

The following are available online at https://www.mdpi.com/2072-6651/12/1/18/s1, Figure S1: Effects of light intensity on minimum fluorescence (F_0_) and maximum fluorescence (F_m_) after addition of different H_2_O_2_ concentrations, Figure S2: Pure microcystin-LR (MC-LR) concentrations 24 h after addition of different H_2_O_2_ concentrations at three different light intensities, Table S1: Photosynthetic yield (Fv/Fm) during the first 4 h after addition of different H_2_O_2_ concentrations and exposure to different light intensities, Table S2: Parameter estimates of the dose-response models.
