# Supplementary material for: Suppressing Cyanobacteria with Hydrogen Peroxide Is More Effective at High Light Intensities
Source: Toxins (Basel). 2019 Dec 31;12(1):18. doi: 10.3390/toxins12010018 (PMC7020451; doi:10.3390/toxins12010018)
Supplement: Supplementary file 1 [file toxins-12-00018-s001.pdf]

Supplementary Materials: Suppressing Cyanobacteria with Hydrogen Peroxide is More Effective at High Light Intensities

Tim Piel, Giovanni Sandrini, Emily White, Tianshuo Xu, J. Merijn Schuurmans, Jef Huisman and Petra M. Visser

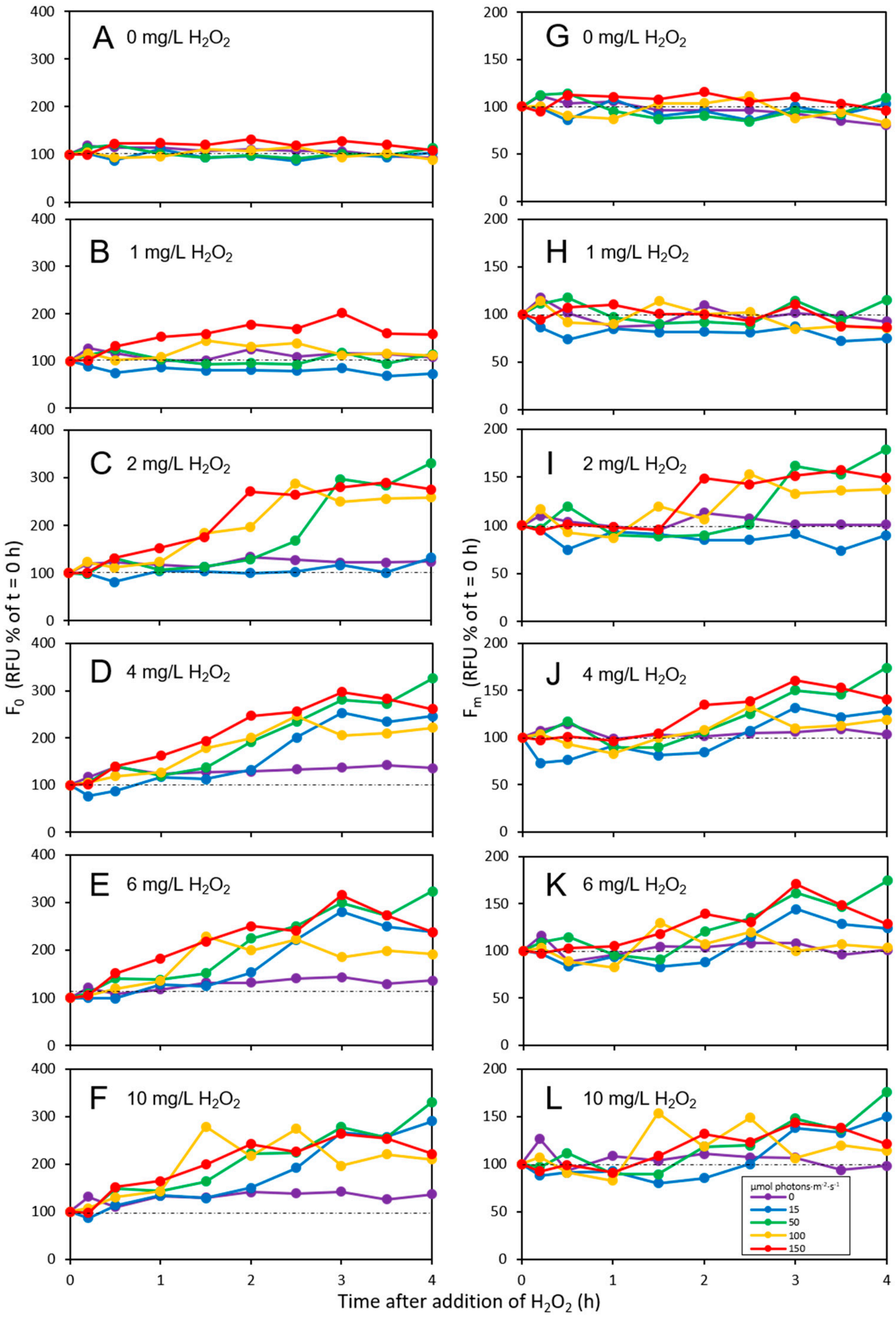

Figure S1. Effects of light intensity on minimum fluorescence (F_0) and maximum fluorescence (F_m) after addition of different H_2O_2 concentrations. The graphs show changes in (**A–F**) F_0 and (**G–L**) F_m of

Microcystis PCC 7806 during the first 4 h after addition of (**A**,**G**) 0 mg L^{-1}, (**B**,**H**) 1 mg L^{-1}, (**C**,**I**) 2 mg L^{-1}, (**D**,**J**) 4 mg L^{-1}, (**E**,**K**) 6 mg L^{-1} and (**F**,**L**) 10 mg L^{-1} of H_2O_2. F_0 and F_m are expressed in relative fluorescence units (RFU), as percentage of the F_0 and F_m value prior to H_2O_2 addition (black dashed lines). F_0 is the minimum fluorescence of a dark-adapted culture (when the reaction centers of PSII are "open") and F_m is the maximum fluorescence after a saturating light pulse (when the reaction centers of PSII are "closed"). Symbols represent the average of two biological replicates; different colours represent different light intensities.

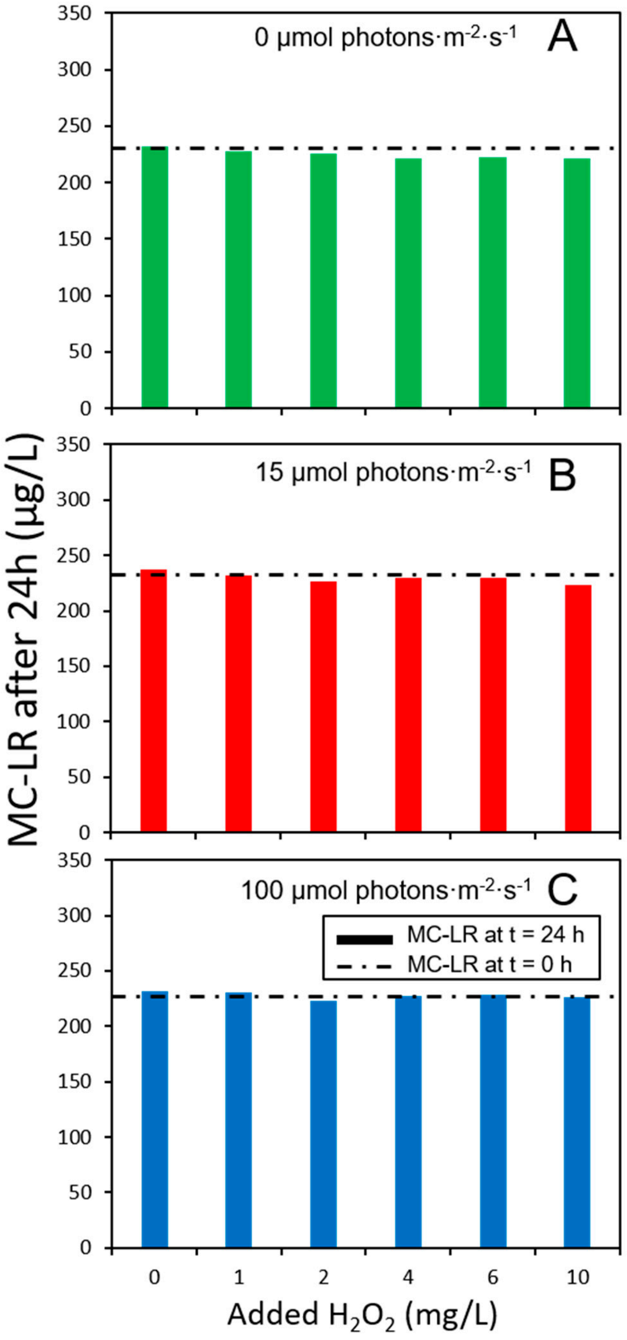

Figure S2. Pure microcystin-LR (MC-LR) concentrations 24 h after addition of different H_2O_2 concentrations at three different light intensities. MC was exposed to (**A**) darkness (0 µmol photons·m^{-2}·s^{-1}), (**B**) 15 µmol photons·m^{-2}·s^{-1}, and (**C**) 100 µmol photons·m^{-2}·s^{-1}. Each bar represents the average of two independent biological replicates per treatment. Dashed lines indicate the MC-LR concentration prior to H_2O_2 addition (t = 0 h).

Table S1. Photosynthetic yield (F_v/F_m) during the first 4 h after addition of different H_2O_2 concentrations and exposure to different light intensities. Each row shows an independent biological replicate; we used two biological replicates per H_2O_2 addition.

Light Intensity	H_2O_2	Time after Addition of H_2O_2 (h)									
(µmol Photons $m^{-2}s^{-1}$)	(mg L^{-1})	0	0.2	0.5	1	1.5	2	2.5	3	3.5	4
	0 (control)	0.434	0.403	0.377	0.374	0.373	0.36	0.359	0.353	0.362	0.354
	0 (control)	0.442	0.402	0.383	0.4	0.367	0.359	0.395	0.352	0.357	0.354
	1	0.446	0.395	0.348	0.36	0.355	0.361	0.358	0.359	0.355	0.348
	1	0.438	0.398	0.361	0.352	0.363	0.366	0.36	0.36	0.354	0.348
	2	0.443	0.397	0.338	0.325	0.335	0.332	0.333	0.325	0.323	0.31
	2	0.442	0.394	0.351	0.353	0.351	0.35	0.346	0.317	0.33	0.321
0	4	0.446	0.395	0.329	0.301	0.296	0.292	0.285	0.28	0.27	0.262
	4	0.438	0.383	0.329	0.305	0.312	0.292	0.287	0.279	0.268	0.267
	6	0.441	0.429	0.307	0.293	0.285	0.277	0.262	0.243	0.237	0.235
	6	0.438	0.406	0.328	0.329	0.304	0.295	0.276	0.271	0.253	0.251
	10	0.437	0.415	0.326	0.305	0.289	0.279	0.262	0.248	0.236	0.212
	10	0.434	0.413	0.332	0.313	0.302	0.285	0.273	0.251	0.247	0.218
	0 (control)	0.475	0.461	0.471	0.467	0.453	0.462	0.477	0.474	0.462	0.471
	0 (control)	0.477	0.467	0.469	0.456	0.467	0.471	0.469	0.472	0.463	0.466
	1	0.477	0.461	0.458	0.473	0.471	0.483	0.487	0.491	0.504	0.488
	1	0.479	0.457	0.485	0.462	0.494	0.487	0.494	0.495	0.505	0.493
	2	0.481	0.447	0.41	0.418	0.406	0.394	0.372	0.333	0.311	0.254
	2	0.479	0.473	0.456	0.426	0.412	0.382	0.368	0.329	0.272	0.217
15	4	0.483	0.471	0.405	0.339	0.283	0.192	0.036	0	0	0
	4	0.478	0.443	0.408	0.328	0.273	0.168	0.024	0	0	0
	6	0.487	0.475	0.374	0.276	0.193	0.06	0.005	0	0	0
	6	0.485	0.466	0.404	0.32	0.261	0.15	0.017	0	0	0
	10	0.49	0.466	0.356	0.258	0.179	0.09	0	0	0	0
	10	0.477	0.514	0.357	0.25	0.164	0.088	0	0	0	0
	0 (control)	0.469	0.459	0.442	0.434	0.434	0.423	0.434	0.438	0.434	0.452
	0 (control)	0.465	0.457	0.439	0.433	0.423	0.421	0.42	0.431	0.432	0.449
	1	0.452	0.45	0.418	0.422	0.437	0.444	0.438	0.437	0.449	0.464
	1	0.458	0.449	0.431	0.417	0.435	0.431	0.433	0.44	0.452	0.456
	2	0.459	0.452	0.409	0.358	0.309	0.226	0.107	0.012	0	0
	2	0.459	0.449	0.408	0.363	0.3	0.224	0.095	0	0	0
50	4	0.47	0.458	0.374	0.284	0.192	0.038	0.004	0	0	0
	4	0.464	0.454	0.356	0.287	0.185	0.038	0.002	0	0	0
	6	0.462	0.451	0.336	0.222	0.093	0	0	0	0	0
	6	0.462	0.465	0.341	0.223	0.112	0	0	0	0	0
	10	0.467	0.454	0.287	0.155	0.03	0	0	0	0	0
	10	0.467	0.451	0.289	0.156	0.022	0	0	0	0	0
	0 (control)	0.465	0.444	0.441	0.429	0.421	0.444	0.435	0.42	0.411	0.416
	0 (control)	0.462	0.435	0.446	0.394	0.422	0.441	0.438	0.427	0.423	0.427
	1	0.452	0.432	0.392	0.351	0.317	0.294	0.26	0.27	0.278	0.286
	1	0.453	0.448	0.394	0.334	0.309	0.284	0.271	0.275	0.275	0.286
	2	0.463	0.445	0.358	0.243	0.217	0.019	0	0	0	0
	2	0.474	0.435	0.364	0.252	0.154	0.023	0	0	0	0
100	4	0.464	0.45	0.316	0.172	0.045	0.006	0	0	0	0
	4	0.462	0.455	0.31	0.175	0.027	0	0	0	0	0
	6	0.467	0.446	0.276	0.111	0.052	0	0	0	0	0
	6	0.459	0.456	0.284	0.124	0.049	0	0	0	0	0
	10	0.452	0.459	0.217	0.06	0.015	0	0	0	0	0
	10	0.464	0.455	0.228	0.059	0.017	0	0	0	0	0
	0 (control)	0.454	0.433	0.421	0.398	0.404	0.388	0.404	0.376	0.36	0.392
	0 (control)	0.466	0.427	0.4	0.397	0.389	0.381	0.382	0.365	0.378	0.384
	1	0.455	0.406	0.324	0.247	0.139	0.021	0	0	0	0
	1	0.449	0.411	0.339	0.246	0.149	0.031	0	0	0	0
	2	0.457	0.432	0.295	0.147	0	0	0	0	0	0
	2	0.462	0.416	0.3	0.171	0	0	0	0	0	0
150	4	0.459	0.443	0.266	0.108	0	0	0	0	0	0
	4	0.464	0.426	0.242	0.083	0	0	0	0	0	0
	6	0.459	0.411	0.206	0.057	0	0	0	0	0	0
	6	0.459	0.424	0.207	0.065	0	0	0	0	0	0
	10	0.456	0.425	0.167	0.012	0	0	0	0	0	0
	10	0.456	0.425	0.164	0.024	0	0	0	0	0	0

Table 2S. Parameter estimates of the dose-response models. The parameters A (EC_{50}) and B (slope) were estimated by fitting the dose-response model to the photosynthetic vitality (PV) data as function of the H_2O_2 concentration using nonlinear regression. The goodness of fit of the models is indicated by the R^2 values and the residual sum of squares (RSS). Part I of the table displays the parameter estimates when the dose-response models are fitted to each light intensity separately. Subsequently, we applied a pairwise comparison to test whether the PV data of two adjacent light intensities are best described by their two separate dose-response models (part II of the table) or by a single dose-response model that captures the PV data of both light intensities (part III of the table). Model selection was based on the Akaike Information Criterion corrected for small sample sizes (AICc). Comparison of part II and part III shows that lower AICc values are obtained when the PV data are described by separate dose-response models for each light intensity. N.A = not applicable, s. a. = see above and refers to the values of A, B, and R^2 as indicated for each light intensity in part I of the table.

Table Part	Light Intensity (μmol Photons·m^{-2}·s^{-1})	# Data Points (n)	# Parameters (k)	Estimated EC_{50} (A)	Estimated Slope (B)	R^2	RSS	AICc
I	0	12	2	13.71	2.41	0.96	104.03	N.A.
	15	12	2	2.00	83.15	1.00	76.06	N.A.
	50	12	2	1.54	122.97	1.00	10.47	N.A.
	100	12	2	1.03	59.71	1.00	0.03	N.A.
	150	12	2	0.30	46.00	1.00	0.00	N.A.
II	0/15	24	4	s. a.	s. a.	s. a.	180.09	20.49
	15/50	24	4	s. a.	s. a.	s. a.	86.53	19.03
	50/100	24	4	s. a.	s. a.	s. a.	10.50	14.81
	100/150	24	4	s. a.	s. a.	s. a.	0.03	3.05
III	0 & 15	24	2	3.82	3.42	0.54	17310.25	24.09
	15 & 50	24	2	1.92	60.23	0.95	2613.08	20.31
	50 & 100	24	2	1.07	58.89	0.98	1175.04	18.71
	100 & 150	24	2	0.97	55.41	0.88	4604.02	21.44
